# Engineering of Synthetic Transcriptional Switches in Yeast

**DOI:** 10.3390/life12040557

**Published:** 2022-04-08

**Authors:** Masahiro Tominaga, Akihiko Kondo, Jun Ishii

**Affiliations:** 1 Engineering Biology Research Center, Kobe University, 1-1 Rokkodai, Nada, Kobe 657-8501, Japan; mtominaga@people.kobe-u.ac.jp (M.T.); akondo@kobe-u.ac.jp (A.K.); 2 Graduate School of Science, Technology and Innovation, Kobe University, 1-1 Rokkodai, Nada, Kobe 657-8501, Japan; 3 Department of Chemical Science and Engineering, Graduate School of Engineering, Kobe University, 1-1 Rokkodai, Nada, Kobe 657-8501, Japan; 4 Center for Sustainable Resource Science, RIKEN, 1-7-22 Suehiro, Tsurumi, Yokohama 230-0045, Japan

**Keywords:** genetic switch, transcriptional switch, yeast, synthetic transcription factor, synthetic promoter, directed evolution

## Abstract

Transcriptional switches can be utilized for many purposes in synthetic biology, including the assembly of complex genetic circuits to achieve sophisticated cellular systems and the construction of biosensors for real-time monitoring of intracellular metabolite concentrations. Although to date such switches have mainly been developed in prokaryotes, those for eukaryotes are increasingly being reported as both rational and random engineering technologies mature. In this review, we describe yeast transcriptional switches with different modes of action and how to alter their properties. We also discuss directed evolution technologies for the rapid and robust construction of yeast transcriptional switches.

## 1. Introduction

The field of synthetic biology has led to the construction of increasingly sophisticated biological systems [1,2], including biosensors to detect viral mRNA [3,4], one of which has been fabricated as a wearable face-mask to detect the novel coronavirus SARS-CoV-2 [4]. The construction of these biological systems necessitates that researchers develop genetic elements, such as promoters and terminators to satisfy the increasing demand for precise control of complex gene expression. In particular, transcriptional switches are required to regulate gene expression in response to intracellular and extracellular stimuli (e.g., metabolites and inducers) and thereby modulate cellular phenotypes via their output [5]. To date, such switches have been utilized to build inducible expression systems [6,7,8,9], genetic circuits [10,11,12,13,14], and metabolite sensors [15,16,17,18,19].

Endogenous systems, such as the Gal4 transcriptional switch [6,7,8] and G-protein coupled receptor systems [20] have long been used as genetic switches in yeast. Alternatively, genetic switches can be artificially created using heterologous, ligand-responsive, DNA binding proteins, exemplified by bacterial transcription factors (TFs), which can be used to control promoter activity. Tet-ON and Tet-OFF systems that regulate gene expression in response to the small molecule, doxycycline (Dox) were first described as synthetic genetic switches for mammalian systems [21] and subsequently used in various fungi [22,23,24]. Yeast genetic switches can also be created at the translational level using aptamers, riboswitches, and ribozymes, as recently reviewed by Ge and Marchisio [25].

Unfortunately, most genetic switches cannot be used directly for synthetic biology applications because of their inappropriate switching properties. For example, such promoters often exhibit detectable activity in the OFF-state (leaky expression), which hampers the regulated high-level expression of toxic proteins [26]. Furthermore, if genetic switches are to be used as elements of a synthetic bioengineering toolbox, they must be designed to respond specifically to the desired target chemical with minimal cross-reactivity, no leaky expression in the OFF-state, and sufficient output when activated (ON-state) to give an adequately large signal-to-noise ratio, as otherwise the construction of complex, higher-order, genetic circuits, especially with layered logic gates, will fail [2].

**Figure 1:**
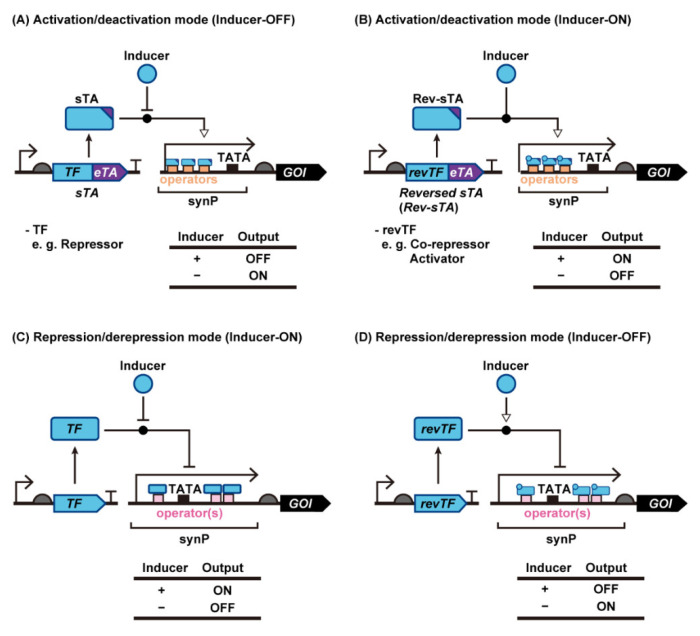
Construction of yeast genetic switches using synthetic transcriptional activators or bare transcription factors. Inducer-ON and inducer-OFF types of genetic switches can be constructed using sTA (**A**,**B**) and intact bacterial repressors (**C**,**D**), respectively. Abbreviations: Core prom, Core promoter; eTA, eukaryotic transcription activator; GOI, gene of interest; revTF, reverse transcription factor; RNAP, RNA polymerase; synP, synthetic promoter; sTA, synthetic transcription activator; TATA, TATA Box; TF, transcription factor.

In the present review, we summarize how synthetic transcription-level genetic switches have been created and improved in yeast. First, we categorize yeast synthetic transcription switches into two groups according to the mode of action: a transcription activation mode (Figure 1A,B), and a transcription repression mode (Figure 1C,D). Then, we describe general and specific strategies to improve the performance of each type of yeast transcriptional switch with regards to the expression ratio between the ON- and OFF-states (i.e., induction fold), inducer sensitivity and specificity, and target promoter specificity. Finally, we present the evolutionary techniques used to improve or create functional genetic switches in yeast, especially in *Saccharomyces cerevisiae*.

## 2. Synthetic Transcriptional Switches with Different Modes of Regulation in Yeast

Synthetic genetic switches in yeast are generally categorized according to two types of regulation (Figure 1). One is an activation mode where transcription from a synthetic promoter (synP) is activated by synthetic transcription activators (sTAs), and the other is a repressor mode where transcriptional inhibition is controlled by transcription repressors. The activator mode is further divided into three groups depending on the mode of action. In the following sections, we describe the mechanism of action of each mode of regulation in detail.

### 2.1. Transcription Activation Mode

Although transcription from a yeast promoter requires the recruitment of multiple endogenous TFs to the promoter, the binding of a single protein fused with eukaryotic transcription activators (eTAs) is sufficient to artificially stimulate the recruitment of yeast TFs. Therefore, synthetic transcriptional switches can be created in yeast by fusing eTAs with ligand-responsive DNA binding proteins, such as bacterial TFs (bTFs; also known as allosterically regulated TFs). Using appropriate design parameters, ligand-dependent binding of bTFs to their operator DNA sequences fused upstream of the yeast core promoter [i.e., a yeast promoter lacking an upstream activation sequence (UAS)] can be translated to the output gene expression [21,24,27,28,29] (Figure 1A,B).

#### 2.1.1. sTAs Based on Bacterial Transcriptional Repressors

The Tet-OFF system [24] is a well-known and proven technology that uses a synthetic transcription activator, i.e., a fusion of tetracycline-responsive transcription repressor TetR and eTA, named VP16. The resultant tetracycline-responsive transcription activator, TetTA, activates transcription from the target synthetic promoter consisting of TetR binding sites (*tetO*) fused upstream with a eukaryotic core promoter based on a yeast *CYC1* promoter which loses its UAS in response to doxycycline, a more effective analog of tetracycline [30]. This system has been used extensively in synthetic biology projects: in particular, this system has been used to explore the function of genes that confer toxic phenotypes because the switches allow regulated expression of the candidate genes [31,32,33].

Bacteria harbor various TetR homologues [34]. The corresponding operator sequences and ligand molecules have been identified for some of these repressor proteins, enabling researchers to construct a series of sTAs and synPs. Ikushima et al. have developed a genetic switch that is tightly controlled by camphor, an inexpensive small molecule [28]. In this case, an sTA was created by fusing three tandem repeats of VP16 and a nuclear localization sequence (NLS) to the CamR transcriptional repressor, a TetR homolog from *Pseudomonas putida*. A corresponding synthetic promoter was also created by embedding six repeats of CamR binding sites (*camO*) between the *ADH1* terminator (to avoid leaky crosstalk from upstream transcription) and the *CYC1* minimal (core) promoter (which lacks the UAS). In the resulting system (the Camphor-OFF switch), the sTA binds *camO* to promote transcription in response to camphor, facilitating camphor-dependent regulation of downstream gene expression via an “inducer-OFF”-type sTA (Figure 1A).

In subsequent research conducted by Ikushima and Boeke [29], a series of “inducer-OFF”-type genetic switches that function in *S*. *cerevisiae* were created using different TetR homolog transcription repressors and their cognate operator DNAs and ligands (Table 1). More recently, the same design was applied to the construction of sensors for malonyl-CoA and xylose in *S*. *cerevisiae* [35]. In addition, the versatility of this strategy was demonstrated by applying it to the construction of a malonyl-CoA sensor in the nonconventional yeast *Komagataella phaffii* [36].

#### 2.1.2. sTAs Based on Bacterial Transcriptional Co-Repressors and Activators

Bacterial transcription repressors can reverse their switching behavior by introducing mutations to them [37]. Thus, opposite regulation can be achieved by using mutant sTAs with these reversed mutations (Figure 1B). The first report of a reversed sTA was a reversed TetTA (rTetTA) based on a reversed TetR mutant that binds to *tetO* in the presence of Dox. The discovery and evolutionary engineering of rTetTA have been reviewed previously [38]. Briefly, rTetTA was first identified following a directed evolution experiment using *Eshcerichia coli* [30] and later was identified in yeast [39]. Dox-sensitivity was subsequently improved using directed evolution in yeast [39] and then using viral evolution [40]. Later, Roney et al. serendipitously discovered a mutation in rTetTA that significantly reduces leaky activation of rTetTA [41]. This system has been used extensively in synthetic biology projects for constructing complex gene circuits [42,43,44]. More recently, using a novel directed evolution platform described below (Section 4.4) [45], a reverse PhlF mutant (rPhlF: PhlF with K86T, Q117R, and E143K) that binds to *phlO* in the presence of 2,4-diacetylphloroglucinol (DAPG) was reported. Furthermore, natural bacterial repressors that bind to their binding sequences upon inducer binding have also be used to construct this type of sTAs; for example, MetJ, which binds to its target sequence *metO* in response to *S*-adenosylmethionine (SAM), was used to construct a SAM-monitoring biosensor in *S*. *cerevisiae* [46].

Ligand-induced DNA binders can also be sought from bacterial transcriptional activators. Moser et al. were the first to describe this type of sTA that can sense methylating compounds, such as methylnitronitrosoguanidine and methyl methanesulfonate, in yeast [47]. In this system, the N-terminal region of the Ada protein from *E*. *coli* was fused with the Gal4 transcription activation domain. Upon addition of methylating compounds, the Ada protein is methylated to bind to the cognate operator sequence, which facilitates transcriptional activation of the synthetic promoter. Subsequently, Castano-Cerezo et al. engineered a 4-hydroxybenzoate-responsive transcription activator, HbaR, from *Rhodopseudomonas palustris* into an sTA by fusing the protein with the transcription activator B112 and the DNA binding protein LexA [48]. Wei et al. reported the fusion of a xylose-responsive transcription activator, XylR from *E*. *coli*, with a eukaryotic transcription activation motif, VPR, or heat shock factor 1 to create a xylose sensor both in *S*. *cerevisiae* and in the oleic yeast *Yarrowia lipolytica* [49]. In a recent example, we described a yeast sTA based on the bacterial quorum-sensor protein LuxTA [45]. Specifically, the TetR-family transcription activator LuxR was fused to three copies of VP16 (VP48) to activate a synthetic promoter in yeast composed of the *GAL1* core promoter fused with 1–10 copies of the LuxR binding sequence (*luxO*), resulting in a genetic switch inducible by the addition of a quorum signal, i.e., 3-oxo-hexanoyl homoserine lactone (HSL).

**Table 1:** Examples of transcriptional switches based on synthetic transcription activators.

Inducer ^a^	bTF	bTF Type ^c^	Source	Additional Motif ^d^	Operators ^e^	CoreP ^f^	Reference
Dox	Reversed TetR (rTetR)	Co-rep	*Escherichia coli*	VP16 × 3	[*tetO*]_7_	*P_GAL1_*_(*Sc*)_	[45]
Dox	rTetR	Co-rep	*E. coli*	VP16 × 3	[*tetO*]_7_	*P_CYC1_*_(*Sc*)_	[39]
Dox	rTetR	Co-rep	*E. coli*	VP16 × 3	[*tetO*]_3 or 4_	*P_GAL1_*_(*Sc*)_	[41]
Dox	TetR	Rep	*E. coli*	VP16ad × 1 or 2	[*tetO*]_1, 2 or 7_	*P_CYC1_*_(*Sc*)_	[24]
DAPG	PhlF	Rep	*Pseudomonas fluorescens*	VP16 × 3	[*phlO*]_7_	*P_CYC1_*_(*Sc*)_	[29]
				NLS, VP16 × 3	[*phlO*]_1_	*P_GAL1_*_(*Sc*)_	[45]
Camphor	CamR	Rep	*P. putida*	NLS, VP16 × 3	[*camO*]_6_	*P_CYC1_*_(*Sc*)_	[28,29]
					[*camO*]_1_	*P_GAL1_*_(*Sc*)_	[45]
Cumate	CymR	Rep	*P. putida*	NLS, VP16 *	[*cymO*]_6_	*P_CYC1_*_(*Sc*)_	[29]
DAPG	Reversed PhlF (rPhlF)	Rep	*P. fluorescens*	NLS, VP16 × 3	[*phlO*]_6_	*P_GAL1_*_(*Sc*)_	[45]
HSL	LuxR	Act	*Vibrio fischeri*	NLS, VP16 × 3	[*luxO*]_1_	*P_GAL1_*_(*Sc*)_	[45]
					[*luxO*]_5_	*P_GAL1_*_(*Sc*)_	
					[*luxO*]_10_	*P_GAL1_*_(*Sc*)_	
SAM	MetJ	Co-Rep	*E. coli*	NLS, B42	[*metO*]_1_	*P_CYC1_*_(*Sc*)_	[46]
Methylating compound	N-Ada ^b^	Act	*E. coli*	GAL4-AD	[*AdaOp*]_1, 3 or 8_	*P_CYC1_*_(*Sc*)_	[47]
Xylose	XylR	Act	*E. coli*	NLS, VPRH	*P_TEF_*_up_-[*Pxo*]_1_	*P_TEF_*_(*Yl*)_	[49]
				NLS, VPRH	[*Pxo*]_1_	*P_TEF_*_(*Yl*)_	
				NLS, VPR	ND	ND	
				NLS, HSF	ND	ND	
				NLS, VPRH	[*Pxo*]_1_	*P_LEU_*_(*Yl*)_	
				NLS, VPRH	[*Pxo*]_1_	*P_YlACC1_*	
				NLS, VPRH	[*Pxo*]_1_	*P_TEF_*_(*Sc*)_	
Malonyl-CoA	FapR	Rep	*Bacillus subtilis*	Prm1	[*fapO*]_1_	*P_AOX1_*_(*Kp*)_	[36]
Benzoate	HbaR	Act	*Rhodopseudomonas palustris*	B112	[LexA binding site]_8_	*P_CYC1_*_(*Sc*)_	[48]

^a^. Doxycycline, Dox; DAPG, 2,4-diacetylphloroglucinol; HSL, homoserine lactone; SAM, S-adenosyl methionine. ^b^. N-Ada, N-terminal 180 amino acids of Ada protein from *E*. *coli*. ^c^. Co-rep, co-repressor; Rep, repressor; Act, activator. ^d^. VP16 and VP16ad, Transcription activation domain of VP16 from herpes simplex virus 2 (VP16: residues from 436 to 447, and VP16ad: residues 367 to 490); NLS, Nuclear localization signal from Simian Vacuolating Virus 40; B42 and B112, transcription activation domain from *E*. *coli*; GAL4-AD, Activation domain of GAL4 (residues from 767 to 881) from *S. cerevisiae*; VPRH, a fusion of 4×VP16 (VP64), a 65 kDa transcription activator domain of human NF-κB (p65), an Rta protein from Epstein–Barr virus, and transactivation domain of human Heat shock factor 1 (HSF); VPR, a fusion of VP64, p65, and Rta; Prm1, transcription activator protein from *Komagataella phaffii*. VP16 *, the number of VP16 repeats is not described. ^e^. The number of transcription factor binding sequences (operators) is shown as a subscript. LexA, a bacterial transcription repressor; *AdaOp*, Ada operator, *P_TEF_*_up_-[*Pxo*]_1_, synthetic hybrid promoter without lacking UAS of *TEF* promoter; *Pxo*, 240-bp promoter sequence including XylR binding sequence from *E*. *coli*. ND, Not described. ^f^. Source organism for each core promoter (coreP) is shown in parenthesis. *Sc*, *S. cerevisiae*; *Yl*, *Yarrowia lipolytica*; *Kp*, *Komagataella phaffii*. ND, Not described.

#### 2.1.3. sTAs Based on Ligand-Dependent Nuclear Localization

**Figure 2:**
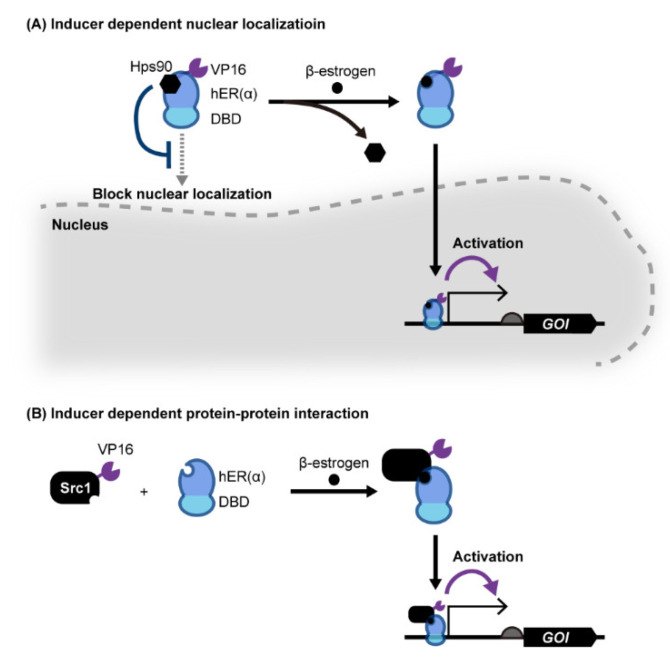
Yeast genetic switches based on eukaryotic ligand-binding proteins. (**A**) The nuclear localization of sTA, which consists of a eukaryotic hormone receptor, a DNA binding domain (DBD) and eTA (VP16), is inhibited by Hsp90p. Upon ligand binding to a hormone receptor, sTA releases from Hsp90p and is localized to the nucleus to activate target promoter transcription. (**B**) Hormone receptor protein hER fused with a DBD binds Src1 only when its agonist hormone is present, activating target gene transcription by recruiting eTA fused with Src1 to the target binding sequence upstream of the promoter. Abbreviations: DBD, DNA binding domain; GOI, gene of interest.

sTAs can also be created with eukaryotic receptor proteins (Figure 2A). In one example, an sTA was created based on human estrogen receptor alpha (hERα) fused with both a DNA binding protein and a transcription activation motif [50,51,52,53,54,55]. In the absence of the native ligand of hERα, β-estrogen (including β-estradiol), the host Hsp90 chaperon complex binds to the sTA and prevents it from being transported to the nucleus (i.e., it remains in the cytosol). Upon ligand binding, the sTA is released from Hsp90 and is transported into the nucleus to bind to the target DNA sequence, resulting in the activation of target gene transcription. More recently, Mormino et al. developed an sTA based on the fusion of an acetic acid-responsive transcription factor from *S*. *cerevisiae*, Haa1, and a DNA binding protein, BM3R1, from *Bacillus megaterium* [56]. In this system, Haa1p is relocated to the nucleus following the binding of acetic acid, which causes binding to the BM3R1 binding sequence fused upstream of the yeast promoter. Using this acetate-responsive genetic switch, it is possible to monitor the acetic acid concentration in yeast within the linear range from 10 to 60 mM. A similar design was used to construct a light-inducible sTA by fusing a photo-sensitive peptide with an eTA, NLS, and a DNA binding protein, where the NLS is concealed from the cellular machinery until light reversibly unfolds the peptide, thereby enabling light-induced nuclear localization of the sTA [57,58].

#### 2.1.4. sTAs Based on Ligand-Induced Protein-Protein Interactions

Recruitment of a transcription activator by using protein-protein interactions has been widely used for yeast two-hybrid (Y2H) systems to investigate protein interactions [59] (Figure 2B). In Y2H systems, eTA and DNA binding domain (DBD) are fused to each protein to be evaluated. This enables reporter gene activation only when these two proteins interact to form a protein complex and thereby recruit the eTA of a nearby promoter of a reporter gene. The Y2H system can be repurposed as a component of a yeast genetic switch using ligand-induced protein-protein interactions. For example, Chockalingam et al. created two fusion proteins: one consists of the ligand binding domain of hERα fused with Gal4 DBD, and the other consists of the mammalian transcription coactivator Src1 fused with the yeast Gal4 activation domain [60]. In this system, hERα and Src1 interact only when estradiol binds to hERα, thereby enabling estradiol-dependent transcription activation. Similarly, light-induced protein dimerization was used to create light-dependent genetic switches in yeast [61,62,63,64]. Furthermore, in addition to sensory proteins, enzymes that bind metabolites and dimerize can be used in this strategy. Chou et al. demonstrated that the endogenous enzymes Idi1p and Erg20p can be repurposed as ligand-responsive dimerizing motifs [65]. In this system, two fusion proteins which consist of an isopentenyl diphosphate (IPP) binding enzyme and a DBD or eTA enable the IPP-dependent co-localization of these fusion proteins onto specific sites of DNA to activate target gene transcription.

#### 2.1.5. Transcription Activation without a Eukaryotic Activation Motif

**Figure 3:**
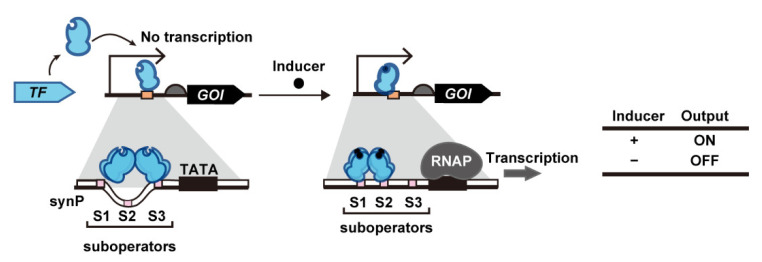
Transcription activation based on intact bacterial transcription activators. Ligand binding to transcription activators induces conformational changes to promoters and the recruitment of RNA polymerase. Abbreviations: GOI, gene of interest; RNAP, RNA polymerase; synP, synthetic promoter TATA, TATA box; TF, transcription factor.

Although most yeast transcription switches use eTAs to activate transcription, some bacterial transcription activators (bTAs) have been recently found to exert transcription activation in *S*. *cerevisiae* even without the fusion of eTA. As the first demonstration of this system, Skjoedt et al. developed biosensors based on bacterial LysR-type transcriptional regulators (LTTR) (Figure 3) [66]. In the native system, the LTTR homotetramer binds to its cognate suboperators [sites 1 and 3 (S1 and S3)] even in the absence of their ligands. Upon ligand binding, the LTTR homotetramer binds to a different set of suboperators [sites 1 and 2 (S1 and S2)], which alters the conformation of the DNA and thereby enhances RNA polymerase (RNAP) binding to the bacterial promoter. In yeast systems, LTTR-binding sequences are embedded in the yeast promoter so that LTTR binding makes the promoter accessible to yeast RNAP. For systematic prototyping, Skjoedt et al. used BenM from *Acinetobacter* sp. ADP1, which responds to *cis*,*cis*-muconic acid (CCM), and its cognate operator DNA, *benO*. Three synthetic promoters were created by fusing the yeast *CYC1* promoter with a single *benO* sequence at different positions. One of the fusions with *benO* upstream of the TATA box resulted in a 20-fold increase in promoter activation upon constitutive expression of BenM from the *TEF1* promoter. Surprisingly, this activation did not require the presence of a eukaryotic activation motif. Cells containing an evolutionarily optimized BenM mutant (H110R, F211V, and Y286N) exhibit a 10-fold induction following the addition of 1.4 mM CCM. This synthetic promoter configuration has been used to construct three different biosensors by using the operators of FdeR (*fdeO*), ArgP (*argO*), MdcR (*mdcO*) and PcaQ (*pcaO*) instead of *benO*; these operators respond to naringenin, L-arginine, and malonic acid and protocatechuic acid, respectively [66,67]. In subsequent research by Snoek et al., the use of fluorescence-activated cell sorting (FACS)-aided directed evolution of BenM enabled the identification of BenM mutants with reversed switching phenotype (CCM-induced deactivation; CCM-OFF), improved induction-fold and operational range, and altered ligand specificity [19].

### 2.2. Transcription Repression Mode

When an intact bacterial transcription repressor binding sequence is fused to a position upstream of the TATA box in a yeast promoter, transcription is blocked by inhibition of RNAP complex binding. Transcription from the resultant synP is derepressed when the ligand-bound bTFs dissociate from the synP. This mode of action has allowed the creation of many inducible yeast promoters.

The earliest example is a tetracycline-inducible system based on TetR and *tetO,* called Tet-ON, in which intact TetR binds to *tetO* and hinders the binding of RNAP to the promoter (transcription repression) [13,68,69]. Following the binding of anhydrotetracycline (aTc) to TetR, TetR dissociates from the operator, resulting in the de-repression of the synthetic promoter (Figure 1C, Table 2). Any transcription repressor and its binding target sequence from bacteria can be assembled into this type of genetic switch. Ikushima and Boeke demonstrated repressor-based sensing using the native PhlF repressor without the VP16 transcriptional activation domain (the DAPG-ON switch) [29]. One or two *phlO* elements were embedded downstream of the constitutive *ADH1* promoter; free PhlF binds to the *phlO* elements (without DAPG), repressing reporter transcription. In the presence of DAPG, DAPG-bound PhlF dissociates from the *phlO* elements, which permits the initiation of reporter transcription. It is also possible to create such synthetic systems using the various inducer-responsive transcription factors XylR [13,70,71,72], FdeR [73], FadR [74], FapR [36,75,76], LacI [13,68,77,78] and VanR [18], which respond to xylose, naringenin, fatty acids, malonyl-CoA, isopropyl-β-D-thiogalactopyranoside (IPTG), and vanillin, respectively. Despite differences in core promoter architecture between yeasts, the same design has been applied to fission yeast [78] and the methylotrophic yeast *K. phaffii* [36,77]. In the latter instance, Cao et al. inserted the LacI binding sequence (*lacO*) downstream of the *GAPDH* promoter, which resulted in a 6-fold induction of gene expression upon the addition of IPTG [77]. In principle, reversed bTF described in Section 2.1.2 can be used to repress target gene expression upon inducer addition (Figure 1D).

## 3. Strategies to Improve the Performance of Yeast Transcriptional Switches

**Figure 4:**
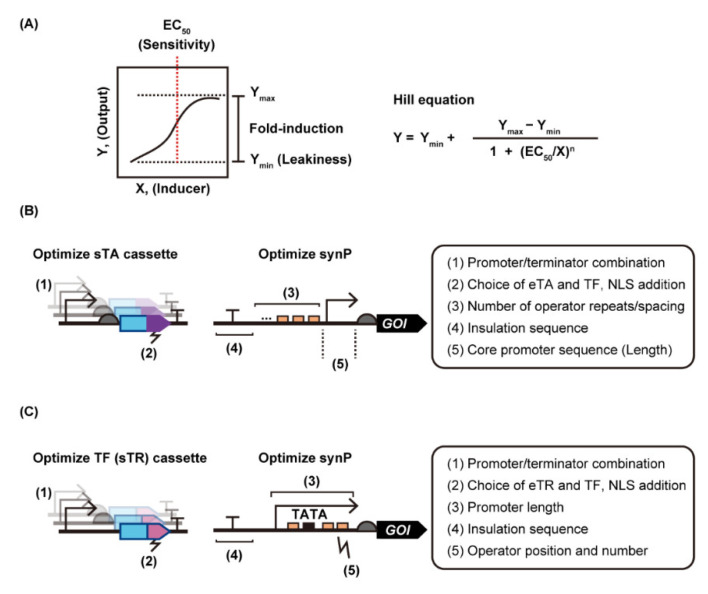
Engineering strategy for yeast genetic switches with different modes of action. (**A**) A dose-response curve for a representative “inducer-ON” switch is shown. To evaluate switching performance, reporter gene expression in the presence of different concentrations of inducer is quantified and the resultant data are fitted to the equation to obtain the response function. Strategies to alter the response function of yeast genetic switches with an activation/deactivation mode (**B**) and repression/de-repression mode (**C**) are illustrated. Abbreviations: eTA, eukaryotic transcription activator; eTR, eukaryotic transcription repressor; GOI, gene of interest; NLS, nuclear localization signal; synP, synthetic promoter; sTA, synthetic transcription activator; sTR, synthetic transcription repressor; TATA, TATA Box; TF, transcription factor.

Any ligand-responsive DNA binding protein and its binding sequence can be assembled with yeast promoters to build synthetic gene regulation systems in yeast; however, the prototype switches often perform poorly. Substantial optimization is required to make the systems practical (Figure 4). Ideally, genetic switches should exhibit strong target gene expression in the ON-state with minimal leakiness in the OFF-state, i.e., a strong induction fold. In addition, sensitivity, i.e., the inducer concentration required to turn gene expression ON (often defined as 50% activation, i.e., EC_50_), is also important, especially for metabolic engineering purposes that require real-time monitoring of intracellular metabolite concentrations, or target gene induction with minimal physiological perturbation caused by large amounts of inducer. We summarize the engineering strategies used to date to improve these properties of yeast transcriptional switches in the following sections.

### 3.1. Strategies for Improving Fold-Induction

In the following subsections, we describe strategies to improve these properties of yeast transcriptional switches. Since the possible strategies are different depending on the mode of action, we will introduce each strategy one by one. Additionally, we describe genetic circuits to improve fold-induction.

#### 3.1.1. sTA-Based Yeast Transcriptional Switches

Since any synP shows substantial basal promoter activity, minimizing basal promoter activity is one of the most important points for constructing yeast transcriptional switches with a large signal-to-noise ratio. First, the basal activity of synP can be modulated by altering the core promoter sequence [52]. Readthrough from upstream transcription can be a source of leakiness from the deactivated synP. This leakiness is often blocked by the addition of a terminator sequence [24,29,55]. In addition to the intrinsic leakiness of the synP, ligand-independent sTA binding can occur when too many operators are fused to synP. Thus, the number of operators should be optimized to maximize sTA activation while minimizing leakiness [52] but optimization is challenging because the optimal number of repeats is different depending on the level of sTA expression. For example, McIsaac et al. reported that the induction-fold of estradiol inducible yeast genetic switches was maximized using four copies of the sTA binding site fused to the core promoter [52]. Too much expression of the sTA increases ligand independent binding to the synP, while gene expression in the ON-state can be reduced if there is insufficient sTA expression. In both cases, the induction fold of the systems decreases. Therefore, the optimal expression level of the sTAs in each system should be screened by using different expression promoters and terminators [41].

The tunability of eTA activity is another important factor for this mode of regulation, but a systematic comparison of eTAs fused to TFs has only recently been reported. In 2020, Qiu et al. compared different eTAs [i.e., Gal4, Med2, VP16, VP64-p65-Rta (VPR) and Med2-Gal4] fused to the FapR repressor in terms of their activation capacities (i.e., gene expression levels in the ON-state) [35]. They found that Med2 outperformed the other motifs in *S. cerevisiae* when fused with FapR. Such strong eTAs can be useful, especially when using TFs with weak DNA binding affinity or when there are difficulties with high-level expression in yeast. Again, screening for the optimal combination of TFs and eTAs is required for each transcriptional switch because effective eTA activity is highly dependent on the DNA binding capacity of the TFs.

#### 3.1.2. LTTR-Based Yeast Transcriptional Switches

Due to a lack of detailed understanding of how native LTTR-family transcriptional activators activate the transcription of eukaryotic promoters, systematic construction of synPs or directed evolution of TFs are the only practical strategies for improving such yeast switches. For synP construction, Ambri et al. screened 106 and 133 promoter designs to identify the optimal positions in synthetic promoters for two different bTF-binding sites (*benO* and *pcaO* for BenM and PcaQ, respectively) at single-nucleotide resolution in *S. cerevisiae* [67]. The optimal insertion position was quite different for these two bTFs, and only a few constructs gave distinct switching behavior. For bTF engineering, Snoek et al. reported that FACS-assisted directed evolution of BenM enabled more than a 15-fold improvement in fold-induction [19].

#### 3.1.3. Repressor-Based Yeast Transcriptional Switches

The choice of yeast promoter and the position of the operator greatly affect both the basal (derepressed) and repressed promoter activities. In particular, appropriate operator positions should be screened at single base-pair resolution [67]. Ambri et al. screened 81 promoter designs to identify the optimal positions in synthetic promoters for VanR-binding sites (*vanO*) at single-nucleotide resolution in *S. cerevisiae*. Notably, the optimal insertion position differed from that of the LysR-type transcription activator [67]. Recently, Chen et al. performed systematic optimization of this type of genetic switch [13]. First, they found that the minimal synthetic promoter sequence with a 20-bp poly(T) sequence located 4 bp upstream of the TATA box had strong activity independent of nutrient conditions. They also found that blocking transcription readthrough by placing appropriate yeast terminators and ribozymes upstream of the regulated promoters could minimize the leakiness of the repressible genetic switches. The tightest repression by bTF was achieved by separating the two operators by more than 20 bp. Using these design strategies, they developed three strongly repressible switches with >100-fold induction and low basal output and applied the resultant genetic switches with a wide dynamic range to automatically assemble yeast genetic circuits.

In addition to massive screening for optimizing the architecture of synthetic promoters, bTF homologs that respond to the same inducer molecules can be screened to construct yeast transcriptional switches with different switching performances; however, differences in the DNA- and ligand-binding affinity and stability of each TF in yeast complicates the optimization process of yeast genetic switches. To date, XylR, FapR, and FadR have been cloned from seven, three and two species, respectively, and evaluated as components of yeast genetic switches to obtain maximum regulation (Table 2). For example, the choice of XylR–operator combinations substantially affect the switching performance of xylose-responsive switches; following xylose addition, maximum fold induction was obtained with a XylR/operator pair from *Bacillus subtilis* [71].

If the repression of intact TFs is insufficient, transcriptional repression of given TFs can be strengthened by fusing chromatin remodeling modules, such as Ssn6 and Tup1. The effectiveness of this method was verified by constructing genetic switches with XylR-Ssn6 [72], TetR-Ssn6 and TetR-Tup1 [23] fusions. Two other eukaryotic repressor domains, Mxi1 and KRAB, which are frequently used as components of CRISPRi systems [79], could be used for this purpose, although their use in the context of inducible synthetic transcription repressors has not been experimentally demonstrated.

#### 3.1.4. Genetic Circuits: Assembly of Different Switches

**Figure 5:**
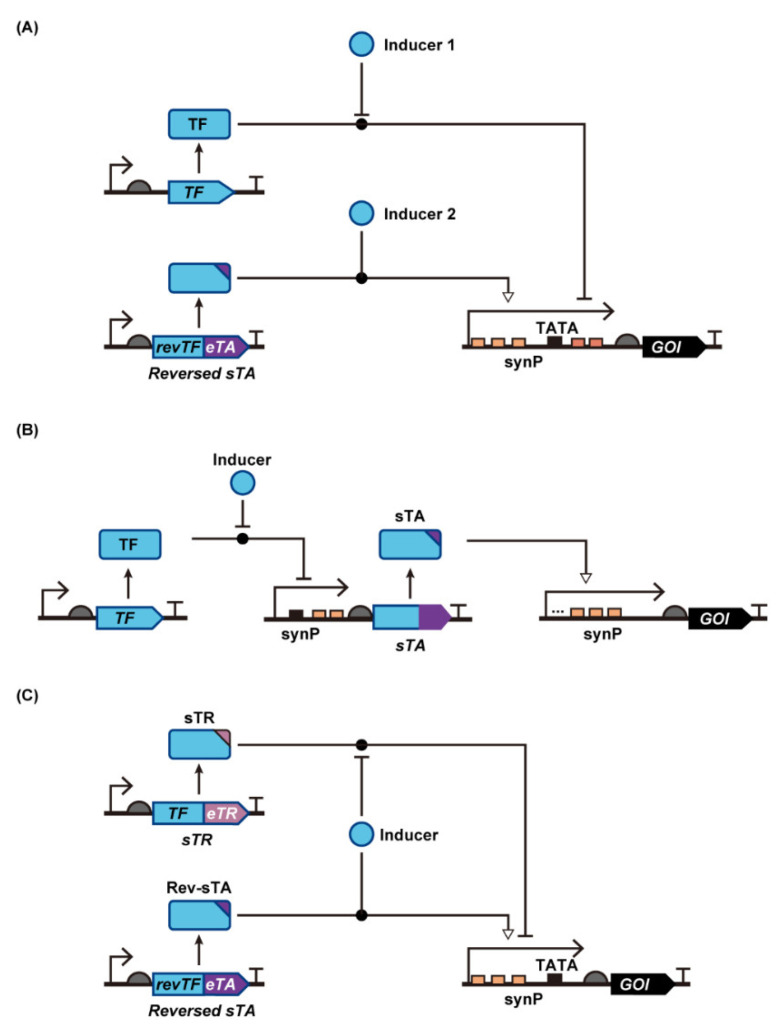
Yeast genetic circuits that increase induction-fold. (**A**) Yeast transcriptional switch that uses both activation/deactivation and repression/de-repression modes of regulation. (**B**). Transcription cascade that amplifies inducer-triggered expression switching. (**C**). Yeast genetic switch that uses both sTA and sTR. Abbreviations: eTA, eukaryotic transcription activator; GOI, gene of interest; synP, synthetic promoter; sTA, synthetic transcription activator; sTR, synthetic transcription repressor; TF, transcription factor.

Transcriptional switches with different modes can be combined to improve and maximize fold-induction by fusing the binding sequence for sTA and intact bTF upstream and downstream of the yeast core promoter, respectively (Figure 5A). For example, Mazumder et al. created a hybrid promoter comprising five upstream binding sequences for testosterone-responsive sTA and a single downstream *lacO* for LacI binding [80]. The resulting system enabled the AND-gated regulation of reporter gene expression: i.e., strong gene expression was induced only when the concentrations of both testosterone and IPTG were sufficient. Another example is a transcription cascade that amplifies ON/OFF regulation (Figure 5B). Recently, Naseri et al. reported LacI/*lacO*-based regulation in combination with an artificial transcription activator based on a plant-derived TF with up to 2020- and 63-fold induction upon IPTG addition in *S. cerevisiae* [81] and *K. phaffii* [82], respectively.

In addition to eTAs, eukaryotic transcription repressors (eTRs) can be fused to bTF to create a synthetic transcription repressor (sTR), which represses synP activity in response to an inducer. By fusing an eTA and eTR to different TFs, yeast genetic switches with dual modes of regulation can be constructed (Figure 5C). The earliest example, by Belli et al., described the simultaneous use of doxycycline (Dox)-responsive sTA and sTR, rTetTA and TetTR, respectively [23]. rTetTA was a fusion of eTA and reversed TetR that binds to *tetO* in the presence of Dox, and rTetTR was a fusion of eTR (Ssn6 or truncated Tup1 from *S. cerevisiae*) and TetR that binds to *tetO* in the absence of Dox. Without Dox, TetTR binds to synP to repress basal promoter activity. Upon Dox addition, rTetTA instead of TetTR binds to synP to activate synP. This dual regulation enabled over 1000-fold induction of reporter gene expression.

### 3.2. Strategy to Modify Inducer Sensitivity and Specificity, and DNA-Binding Specificity

Unlike fold-induction, inducer sensitivity [i.e., EC_50_ (Figure 4A)] and specificity are solely dependent on the binding affinity between TF and its inducer molecule or operator DNA. Thus, these factors can be modulated only by mining different transcription factors derived from other species, by performing protein engineering, or by directed evolution. For example, the xylose-responsive transcription factor XylR has been extensively mined [13,49,70,71,72] (Table 1 and Table 2). Of the 8 XylR homologs tested, XylR from *C. crescentus* exhibited the highest sensitivity to xylose, enabling the detection of as low as 2 µM xylose [72].

**Figure 6:**
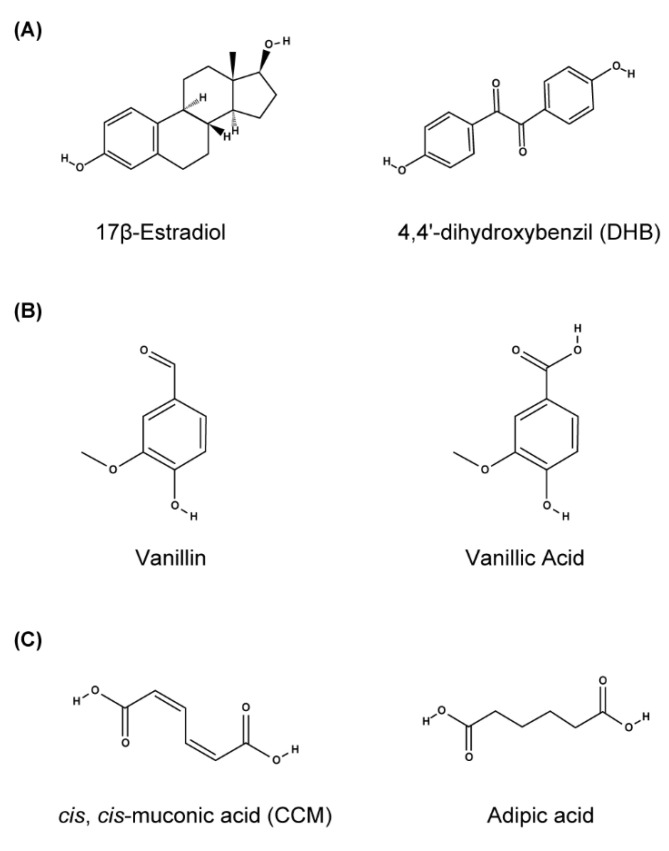
Chemical structure of inducers for wild-type and mutant hERα (**A**), VanR (**B**), and BenM (**C**).

Alternatively, directed evolution is a promising methodology to alter inducer sensitivity and the specificity of TFs. For example, a 17β-estradiol-responsive human receptor was converted into a mutant receptor responsive to the synthetic nonsteroidal compound 4′-4′-dihydroxybenzyl (DHB) [60] or to synthetic ligands [83] with the aid of directed evolution using genetic selections (see also Section 4.3). Evolutionary engineering has also been applied in yeast to improve the sensitivity of rTetTA to Dox [39] and LuxTA to HSL [45] and to alter the specificity of BenM (from CCM to adipic acid) [19] and VanR (from vanillic acid to vanillin) [18] (Figure 6).

Unlike responsivity to inducers, DNA binding specificity can be easily altered by changing the DNA-binding domain (DBD) of eukaryotic ligand-binding proteins to those of other DNA binding proteins. For example, estradiol-inducible synthetic transcription factor, described in Section 2.1.4, can be altered to recognize different DNA sequences by replacing GAL4 DBD with the sequence for *E. coli* LexA protein [55]. DNA binding specificity can be rationally engineered by using a four-finger zinc-finger array to relieve the design constraint of the binding sequence [52]. Such a “module-swapping strategy” can be successfully applied to bacterial transcription factors [84,85,86], although the resultant chimeric TFs have not been used in yeast.

## 4. Directed Evolution of Transcriptional Switches in Yeast

Directed evolution is a practical strategy to develop useful transcriptional switches in yeast, where switch variants with desired performances are designed to be selected from a library with randomized components. This strategy has been widely used in prokaryotic systems, especially in *E. coli*, resulting in a large genetic switch toolbox. In contrast, only a few examples of this evolutionary engineering strategy have been reported in yeast [1,87,88,89,90,91,92,93], although the tuning process of eukaryotic genetic switches is far more difficult than the process required for prokaryotic cells. For example, the behavior of eukaryotic promoters is easily affected in an unpredictable manner by changes to the surrounding sequences [94,95]. In the following subsections, we summarize recent successes in the evolutionary engineering of genetic switches in yeast, as well as the methodologies used in each example.

### 4.1. Fluorescence-Based Screening

Most genetic switches have been evaluated by placing genes for fluorescent proteins to provide an output. Fluorescence measurements are then conducted using multiwell-based plate readers or flow cytometry. Ellis et al. developed a tetracycline-responsive genetic switch in yeast by fusing the *GAL1* promoter with two distinct *tetO* sequences and one *lacO* sequence, where the TetR and LacI protein binds to repress transcription from the *GAL1* promoter [68]. They found switch variants with optimal characteristics for the construction of different genetic circuits (a feed-forward loop and timer) by performing green fluorescent protein (GFP)-based screening of synthetic promoter variants in which the sequences around the operators were randomly mutated. Urlinger et al. performed GFP-based screening of approximately 1000 clones on plates with and without Dox to identify TetTA mutants with reversed phenotypes [39]. They also performed a second round of mutagenesis and screening to identify rTetTA mutants with improved sensitivity. As discussed in a previous review article [96], GFP-based colorimetric screening is particularly important when the cells show clonal populations. Flow-cytometry-based screening, rather than agar-plate or plate reader-based screening, can be essential.

### 4.2. ON/OFF Selections Using FACS

FACS enables the screening of libraries with desired output levels (i.e., fluorescence intensity) at given conditions of as many as 10^8^ variants per day [97,98,99]. This allows for a high-throughput selection of vast libraries of genetic switches. Using a FACS-based selection strategy, directed evolution of the responsivity of bacterial activators that are responsive to muconate (BenM) [19,66] and vanillin (VanR) [18] was performed to obtain mutants with improved specifications in *S. cerevisiae*. Because FACS-based selection can be performed with tunable selection thresholds by changing the gating conditions, it is independent of the specifications of prototype sensors. However, selection efficiency is highly dependent on both the selection and gating conditions. Thus, sorting experiments must be repeated until the correct selection/gating condition is achieved, given the lack of *a priori* knowledge related to the necessary selection conditions for each system.

### 4.3. ON/OFF Selections Using Genetic Selections

Positive and negative selection can be used to enrich genetic switches with defined outputs under ON and OFF conditions. Positive selection markers include auxotrophic markers [e.g., His3/3-aminotriazole (3-AT)] [100], antibiotics markers (e.g., Ble/Zeocin) [101], and counter-selectable markers [102], such as Ura3/5-fluoroorotic acid (5-FOA) [100] and herpes simplex virus thymidine kinase (hsvTK)/5-fluorodeoxyuridine (5FdU) [103]. Directed evolution of genetic switches using genetic selection was first demonstrated by Chockalingam et al., who converted a 17β-estradiol responsive human receptor into a synthetic nonsteroidal compound receptor, namely a DHB-responsive receptor [60]. Only cells with the synthetic transcription activator based on the mutant hormone receptor that responded to DHB grew on media lacking histidine in the presence of DHB by expressing significant amounts of HIS3. Four rounds of saturation mutagenesis to the ligand binding domain and one round of whole-gene mutagenesis followed by genetic selection and screening enabled the authors to identify the mutant that was specifically responsive to DHB. Later, Klauser et al. demonstrated the use of both ON/OFF selections to enrich functional translational switches [100]. In their system, the product of *HIS3*, imidazole glycerol phosphate dehydratase, was gradually inhibited depending on the concentration of its inhibitor, 3-AT. Titration of 3-AT at different concentrations enabled the enrichment of cells with higher outputs in the ON-state (ON selection). The OFF selection was based on *URA3*, coding orotidine 5-phosphate decarboxylase. In the presence of 5-FOA, cells expressing more *URA3* converted more 5-FOA into the toxic compound 5-fluorouracil, which enabled the enrichment of cells without leaky *URA3* expression in the absence of the ligand.

### 4.4. “Screening of Selection” Strategy

Despite the successes of evolutionary strategies described above, this strategy has not been widely applied to genetic switches in yeast. As previously described [96], stringent selection can be performed when the genetic switches to be selected outcompete other nonfunctional (always-ON and always-OFF) variants; however, it is more difficult to select for mutants with a distinct ON-state output from the majority of variants with slightly weaker output or *vice versa*. In most cases, even low expression levels of ON and OFF selection markers are sufficient to allow cell growth and to cause cell death, respectively, which results in low selection efficiency. Moreover, genetic selection fails to discriminate desired genetic switches from others when their output levels under selection are too far from the given selection threshold [100,104]. Thus, it is important to identify the appropriate selection conditions that selectively enrich rare mutants with slightly improved performance. To meet this challenge, researchers must reconstruct the selector systems or repeat the sorting experiments in almost each directed evolution cycle until appropriate selection conditions are obtained.

One possible solution to this problem would involve performing multiple selections for different selection pressures in parallel and choosing the most promising selection pools from which the improved mutants would be screened (i.e., “screening of selection conditions”). We recently described an evolutionary platform for yeast genetic switches that could meet this demand using a trifunctional fusion protein consisting of hsvTK, Zeocin-resistance protein (Ble), and GFP [45]. This fusion protein, hsvTK-Ble-GFP, facilitates seamless ON/OFF selection with different selection pressures and requires only liquid handling in parallel using multiwell plates. The ON/OFF-selected cell populations can be characterized seamlessly using flow cytometry, identifying promising populations, from which improved genetic switch mutants can be screened. As a demonstration of this platform, 14 different selection conditions were tested in parallel from which two promising conditions were subjected to further screening to successfully identify improved Dox-ON switches.

## 5. Conclusions

As described in this review, a number of bacterial transcription factors (TFs) can easily be assembled into yeast genetic switches with or without the use of an evolutionary strategy. This method is becoming the gold-standard for the engineering of genetic switches in yeast; however, rational engineering for the functional tuning of each component, which is crucial for maximizing genetic switch performance, is not currently feasible. An evolutionary platform for genetic switches in yeast was recently demonstrated [18,45,66] and thus, the number of such synthetic switches has rapidly increased, highlighting the potential of prokaryotic TFs as an untapped resource for yeast genetic switches. Umeno et al. argued that TFs are intrinsically evolvable; therefore, mutants that are responsive to non-native compounds can be attained within a few directed evolution cycles [37]. Indeed, dozens of new or evolved genetic switches (biosensors) based on bacterial TFs have been reported within the last 3 years [1,105,106,107,108,109,110,111,112,113]. Library creation guided by machine-learning technology, combined with deep mutagenesis and extensive sequencing technology, will further accelerate the discovery of novel TF mutants [113]. TFs created in this manner will be applied to yeast and then further evolved in yeast, possibly with the aid of in vivo autonomous mutagenesis techniques [114,115,116] as well as automated continuous evolution technologies [117].

Genetic switches in yeast that can sense intracellular metabolites, i.e., metabolite sensors, are beginning to be used in the high-throughput screening of higher metabolite-producing cells [75,118] and in the metabolite-responsive dynamic control of enzyme expression in yeast (see recent reviews [119,120]). These applications require that genetic switches be optimized (evolved) depending on their purpose. For example, the continuous evolution of *cis*,*cis*-muconic acid production in yeast uses the evolved sensory protein BenM [120]. Furthermore, because yeast genetic switches and their assembly (genetic circuits) are based on bacterial TFs optimized in yeast, they are readily transferable into mammalian systems [121,122,123]. Expansion of the well-optimized (evolved) yeast genetic switches reviewed above will result in the accelerated development of more complex mammalian systems. Taken together, directed evolution platforms for genetic switches in yeast may be a key technology for advancing eukaryotic synthetic biology.

## Figures and Tables

**Table 2:** Examples of yeast transcriptional switches based on bacterial repressors.

Inducer ^a^	bTF	Source of bTF and Operator	Additional Motif ^b^	Operators	Yeast Promoter ^d^	Reference
aTc	TetR	*Esherichia coli*	NLS	[*tetO*]_2_	*P_tet_*_(_*_Sc_*_)_ ^e^	[13]
aTc	TetR	*E. coli*	–	[*tetO*]_2_	*P_Lib_*_T_ ^f^	[68]
aTc	TetR	*E. coli*	–	[*tetO*]_2_	*P_PFY1_*_(*Sc*)_	[69]
DAPG	PhlF	*Pseudomonas fluorescens*	NLS	[*phlO*]_1 or 2_	*P_ADH1_*_(_ *_Sc_*_)_	[29]
Vanilic acid	VanR	*Caulobacter crescentus*	–	[*vanO*]_2_	*P_TEF1_*_(_ *_Sc_*_) or_ *P_CYC1_*_(_ *_Sc_*_)_	[18]
Vanillin						
Naringenin	FdeR	*Herbaspirillum seropedicae*	NLS	[*fdeO*]_1_	*P_GPM1_* _(_*_Sc_*_)_	[73]
Fatty acids	FadR	*E. coli*	NLS	[*fadBA_EC*]_1 or 3_	*P_GAL1_* _(_*_Sc_*_)_	[74]
		*Vibrio cholerae*	–	[*fadBA_VC*]_1 or 3_		
Malonyl-CoA	FapR	*Bacillus subtilis*	Prm1	[*fapO*]_1–3_	*P_GAP_* _(_*_Kp_*_)_	[36]
		*B. subtilis*	NLS	[*fapO*]_1 or 2_	*P_GPM1_* _(_*_Sc_*_)_	[76]
		*B. subtilis*	NLS	[*fapO*]_1–3_	*P_TEF1_* _(_*_Sc_*_)_	[75]
Xylose	XylR	*B. licheniformis*	NLS	[*xylO*]_2_	*P_xyl_*_(_ *_Sc_*_)_^e^	[13]
		*B. subtilis*				
		*Tetragenococcus halophile*				
		*Clostridium difficile*				
		*Lactobacillus pentosus*				
		*Caulobacter crescentus*				
		*T. halophile*	NLS	[*xylO*]_1_	*UEE_TEF1-_P_GAL1m_*_(_*_Sc_*_)_ ^g^	[70]
		*C. difficile*	NLS	[*xylO*]_1_	*UEE_TEF1-_P_GAL1m_*_(_*_Sc_*_)_ ^g^	
		*L. pentosus*	NLS	[*xylO*]_1_	*UEE_TEF1-_P_GAL1m_*_(_*_Sc_*_)_ ^g^	
		*C. crescentus*	NLS, Ssn6	[*xylO*]_1 or 2_	*P_TEF_*_(*Ag*)_	[72]
		*Staphylococcus xylosus*	NLS	[*xylO*]_1 or 2_^c^	*P_GPM1_* _(_*_Sc_*_)_	[71]
		*B. licheniformis*	NLS	[*xylO*]_1 or 2_^c^	*P_GPM1_* _(_*_Sc_*_)_	
		*B. subtilis*	NLS	[*xylO*]_1 or 2_^c^	*P_GPM1_* _(_*_Sc_*_)_	
IPTG	LacI	*E. coli*	NLS	[*lacO*]_2_	*P_lac_* _(_*_Sc_*_)_^e^	[13]
			NLS	[*lacO*]_1_	*P_nmt_*_(*Szp*)_	[78]
			–	[*lacO*]_2_	*P_GAP_*_(*Kp*)_	[77]
			NLS	[*lacO*]_1_	*P_LibL_* ^f^	[68]

^a^. Anhydrotetracycline hydrochloride, aTc; DAPG, 2,4-diacetylphloroglucinol; IPTG, isopropyl-β-D(-)-thiogalactopyranoside. ^b^. NLS, Nuclear localization signal from Simian Vacuolating Virus 40; Ssn6, Transcriptional co-repressor from *S*. *cerevisiae*; Prm1, transcription activator protein from *Komagataella phaffii*. ^c^. Four types of operator positions were evaluated: downstream of TATA box, downstream of UAS, upstream of TATA box, both downstream of UAS and upstream of TATA box. ^d^. Source organism for each yeast promoter is shown in parenthesis. Sc, *S. cerevisiae*; *Kp*, *Komagataella phaffii; Szp*, *Schizosaccharomyces pombe; Ag, Ashbya gossypii*. ^e^. *P_tet_*, *P_xyl_*, and *P_lac_* are completely synthetic promoters, where two operator sequences are separated by a poly (T) sequence, followed by the transcription start site. ^f^. *P_Lib_*_T_ and *P_Lib_*_L_ are a completely synthetic promoter, where two *tetO* sequences are flanked by the randomized sequences. ^g^. Hybrid promotor consisting of the upstream enhancer element of *TEF1* promoter (*UEE_TEF1_*) from *S. cerevisiae* and modified *GAL1* core promoter (*P_GAL1m_*) from *S. cerevisiae* was used.

## Data Availability

Not applicable.
